# Yellow coloured mats from lava tubes of La Palma (Canary Islands, Spain) are dominated by metabolically active Actinobacteria

**DOI:** 10.1038/s41598-018-20393-2

**Published:** 2018-01-31

**Authors:** Jose L. Gonzalez-Pimentel, Ana Z. Miller, Valme Jurado, Leonila Laiz, Manuel F. C. Pereira, Cesareo Saiz-Jimenez

**Affiliations:** 1 Instituto de Recursos Naturales y Agrobiologia de Sevilla (IRNAS-CSIC), Avenida Reina Mercedes 10, 41012 Sevilla, Spain; 2 CERENA, Instituto Superior Técnico, Universidade de Lisboa, Avenida Rovisco Pais 1, 1049-001 Lisbon, Portugal

## Abstract

Microbial diversity in lava tubes from Canary Islands (Spain) has never been explored thus far offering a unique opportunity to study subsurface microbiology. Abundant yellow coloured mats developing on coralloid speleothems in a lava tube from La Palma Islands were studied by next-generation sequencing and DNA/RNA clone library analyses for investigating both total and metabolically active bacteria. In addition, morphological and mineralogical characterization was performed by field emission scanning electron microscopy (FESEM), micro-computed tomography, X-ray diffraction and infrared spectroscopy to contextualize sequence data. This approach showed that the coralloid speleothems consist of banded siliceous stalactites composed of opal-A and hydrated halloysite. Analytical pyrolysis was also conducted to infer the possible origin of cave wall pigmentation, revealing that lignin degradation compounds can contribute to speleothem colour. Our RNA-based study showed for the first time that members of the phylum *Actinobacteria*, with 55% of the clones belonging to *Euzebyales* order, were metabolically active components of yellow mats. In contrast, the DNA clone library revealed that around 45% of clones were affiliated to *Proteobacteria*. Composition of microbial phyla obtained by NGS reinforced the DNA clone library data at the phylum level, in which *Proteobacteria* was the most abundant phylum followed by *Actinobacteria*.

## Introduction

Microbial mats frequently coat extensive areas of walls and ceilings of karstic caves and lava tubes, usually developing yellow, tan, orange, grey, pink and white-coloured biofilms^1–6^. Caves, in general, are featured by constant temperature, humidity and carbon dioxide (CO_2_) the year round, as well as absence of light and scarcity of nutrients forcing microorganisms to adapt their metabolism for surviving in these extreme conditions^7^. The low input of nitrogen, carbon and phosphorus, and the rock chemistry composition of lava tubes have a direct impact on community diversity and adaptation to these habitats. Sulfur, organic carbon and nitrogen have been associated with the chemical composition of basaltic substrates in lava tubes^5,8^. Therefore sulfur and nitrate oxidizers or reducers are common in these environments, such as bacteria from *Proteobacteria* and *Nitrospirae* phyla. Moreover, the input of organic matter from the topsoil through water percolation and the penetration of plant roots into the caves are important nutrient sources for heterotrophic bacteria^9–12^. Different researches based on the study of bacterial communities demonstrated the prevalence of several phylogenetic groups at higher taxonomic levels (e.g. phylum and class) in lava tubes around the world^5,13,14^. The authors reported *Actinobacteria*, *Proteobacteria*, *Acidobacteria*, *Nitrospirae*, *Firmicutes*, *Bacteroidetes* and *Chloroflexi* as the main bacterial phyla present in lava tubes. Species affiliated to the phylum *Actinobacteria* have taken special relevance in recent years owing to the production of primary and secondary metabolites with a potential use in the biotechnological industry^15^. Moreover, the evidence of lava tube formation on Mars has prompted the study of microbial life in lava caves on Earth, propelling geomicrobiology of subsurface environments into the scientific spotlight^1,16^.

The appearance of coloured microbial mats on rock art caves producing biodeterioration awakened interest for studying these communities^2,17,18^. In recent years, study efforts have been focused on trying to explain the origin of coloured biogenic formations as well as the direct relationship between pigmentation and its production by specific phylogenetic groups^4,13,19^. In fact, several studies have been focused on determining bacterial phylotypes as the cause of pigmentation^4,13,14,19^. According to these investigations, the phylum *Actinobacteria* seemed to be critical in these coloured communities, being *Pseudonocardiacea* the potential producers of yellow pigmentation^4,14^. Also, *Gammaproteobacteria* and *Nitrospirae*, represented by *Xanthomonadales* and *Nitrospira*, respectively, could play a functional role in cave pigmentation. However, Hathaway *et al*.^5^ and Riquelme *et al*.^13^ did not find a direct relation between some specific phylotypes and pigment production. These studies have led to a contradictory discussion about mat colouration. Porca *et al*.^4^ observed that the presence of members from the order *Xanthomonadales* and suborder *Pseudonocardinae* as well as *Steroidobacter denitrificans* could have caused yellow pigmentation on karstic cave walls. Lavoie *et al*.^14^ studying lava tubes, pointed that *Actinobacteria* was the main phylotype that produced pigmentation.

In this study we aimed at providing for the first time a comparative assessment of bacterial communities which form yellow mats in a lava tube from La Palma (Canary Islands, Spain). An exhaustive identification has been performed based on next-generation sequencing (NGS) and DNA/RNA clone library analyses for investigating both total and metabolically active bacteria. These analyses can be useful for a better understanding of the bacterial diversity thriving in lava tubes and elucidate on the presence of specific phylogenetic groups causing cave wall pigmentation. In addition, a comparison between the 16S rRNA sequences retrieved from the Azores lava tubes studied by Riquelme *et al*.^13^ and the clone sequences obtained in this study was carried out.

## Results

### Morphological and microstructural features of coralloid speleothems coated with yellow microbial mats

**Figure 1:**
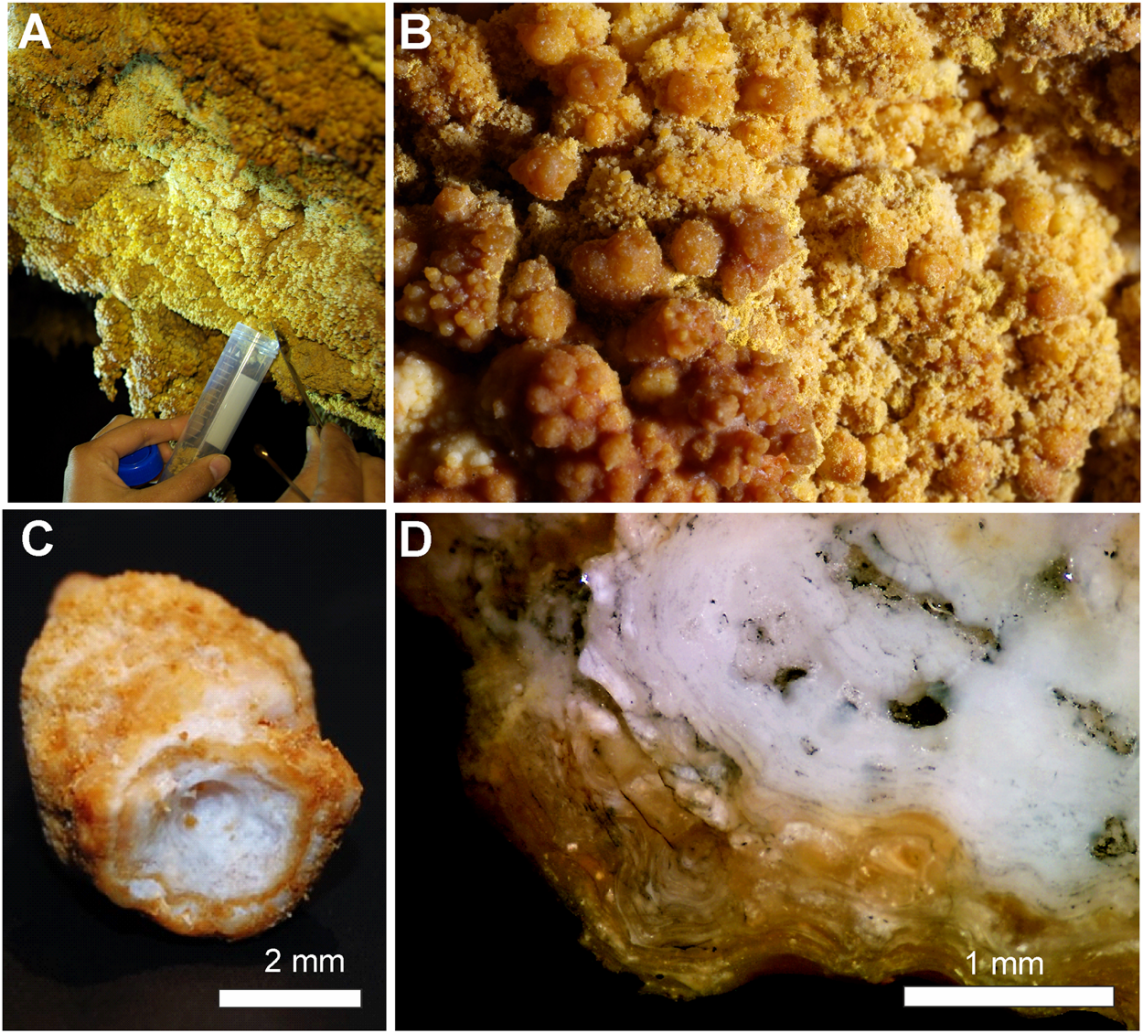
Field images of yellow microbial mats collected in EP01 lava tube from La Palma (Canary Islands). (**A**,**B**) Colonies with a spongy-like texture coating yellowish coralloid speleothems. (**C**) Stereomicroscopy image of a single coralloid speleothem. (**D**) Stereomicroscopy image of a polished cross-section of a coralloid showing the white inner part and yellowish outermost layers of the speleothem.

**Figure 2:**
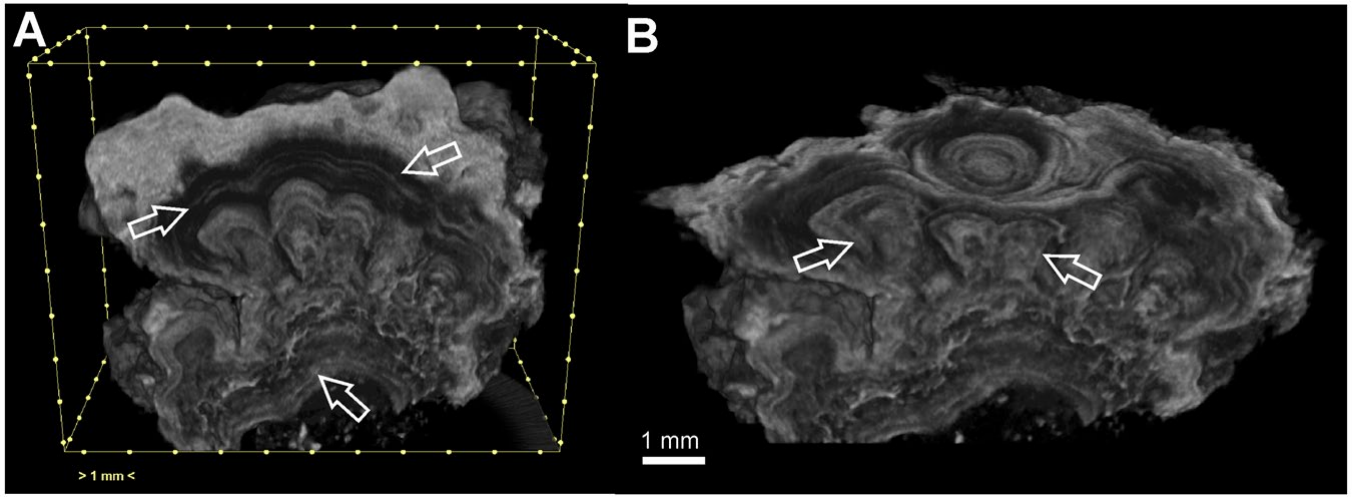
X-ray micro-computed tomography (micro-CT) images of the yellowish coralloid speleothems from EP01 lava tube in La Palma (Canary Islands). (**A**) Fine growth layers (arrows) and variations in material density. (**B**) Development of several branches in the inner part of the coralloid sample (arrows).

Samples of yellow microbial mats were collected from *Honda del Bejenado* (EP01) lava tube in La Palma, Canary Islands, Spain (Supplementary Fig. S1). In this cave, located in a protected natural ecosystem, abundant yellow colonies with a spongy-like texture were observed coating yellowish coralloid speleothems (Fig. 1A,B). These coralloids consisted of small stalactites rarely exceeding 1 cm in size (Fig. 1B,C). A rough surface with yellowish globular aggregates forms the outer zone, whereas a white porous structure forms the inner part of the speleothems (Fig. 1C). The polished cross-section clearly shows the yellowish outer layers of the coralloids and a vitreous and finely lamination in the inner zone (Fig. 1D). Results of micro-computed tomography (micro-CT), a non-destructive technique for imaging the internal structure of solid objects, confirmed the inner layering of the speleothems by the different opacity of the laminae to X-rays for all radiographed coralloid samples (Fig. 2). Greyscale variations correspond to relative X-ray attenuation as a function of elemental composition and density^20^, with bright-coloured areas representing denser material. The micro-CT study revealed a complex internal structure mainly characterized by variations in material density, evidencing changes during speleothem growth (Fig. 2). A very fine layering is observed during the first and final stages of speleothem formation (Fig. 2A). The innermost part of the coralloids is characterized by the development of several branches that evolved independently, which were covered with subsequent fine layers (Fig. 2A). These growth layers are alternated with less compact material (dark grey). Micro-CT results were correlated with field emission scanning electron microscopy combined with energy dispersive X-ray spectroscopy (FESEM-EDS). FESEM-EDS analysis confirmed that differences in opacity observed in the different parts of the coralloid are not due to significant compositional differences, but to textural differences (Supplementary Fig. S2). Element distribution images of a polished cross-section showed a remarkable compositional homogeneity rich in silica (Supplementary Fig. S2A–D), with minor Al and Mg (Supplementary Fig. S2E,F). These latter are particularly detected towards the outermost part of the sample.

**Figure 3:**
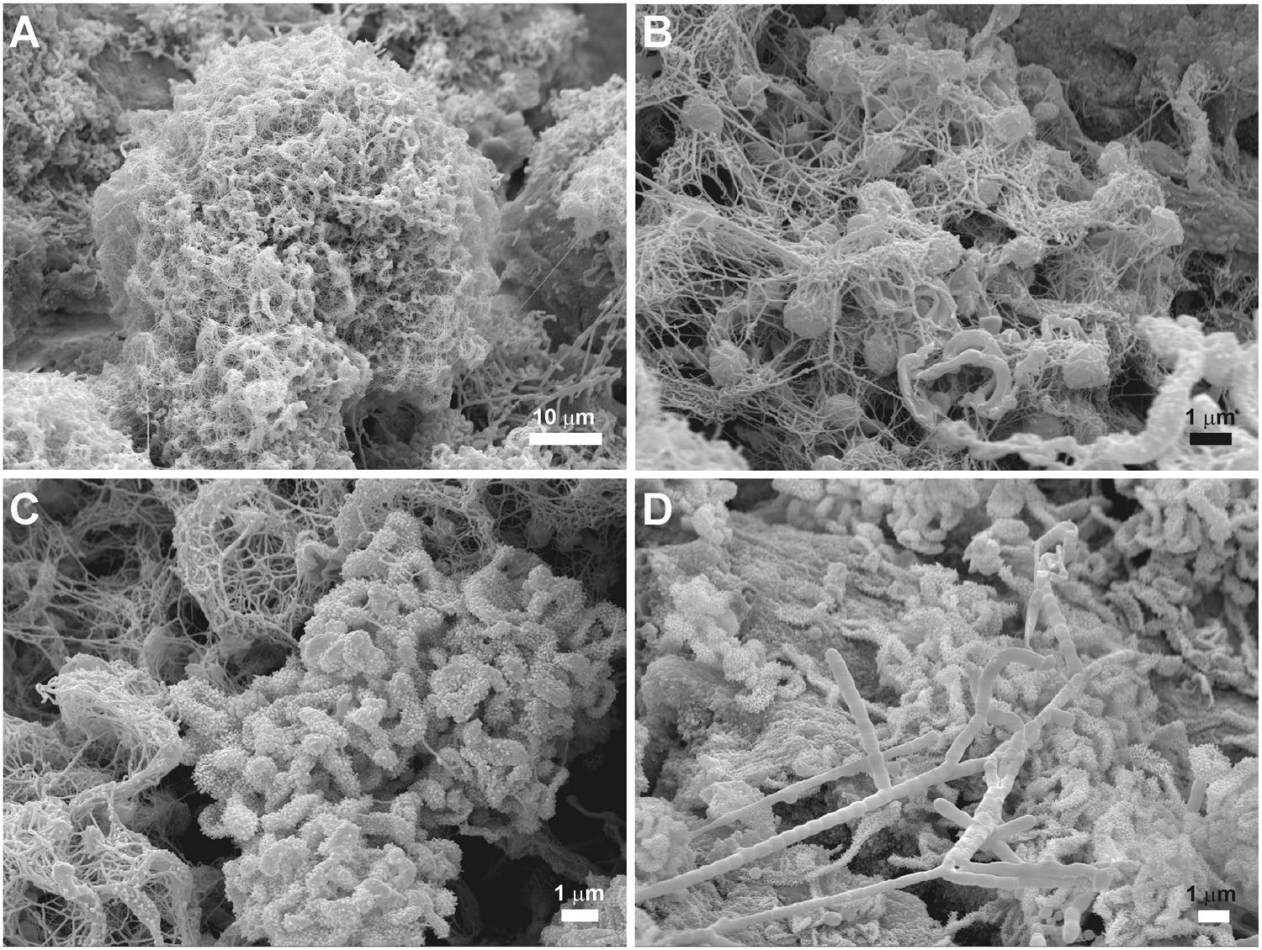
Field emission scanning electron microscopy images of yellow microbial mats from EP01 lava tube in La Palma (Canary Islands). (**A**) Clusters of *Actinobacteria*-like spores intermingled with filamentous forms. (**B**) Detailed image of *Actinobacteria*-like spores intermixed with filamentous forms. (**C**) Spores with profuse surface appendages. (**D**) Arthrospores probably from *Streptomyces*.

FESEM observations of the sample surface revealed a variety of filamentous, spore-producing bacteria spread all over the sample (Fig. 3). *Actinobacteria*-like spores (<1 µm) are produced in dense clusters intermingled with filamentous forms (Fig. 3A–C). Occasionally, these clusters form spheres with a diameter of about 50 µm with lumpy surface composed of a network of hairy filaments and spores. They exhibit smooth surface ornamentation or profuse surface appendages (Fig. 3B,C). Arthrospores probably from *Streptomyces* with true branching are observed in Fig. 3D.

### Mineralogical and geochemical composition of coralloid speleothems

**Figure 4:**
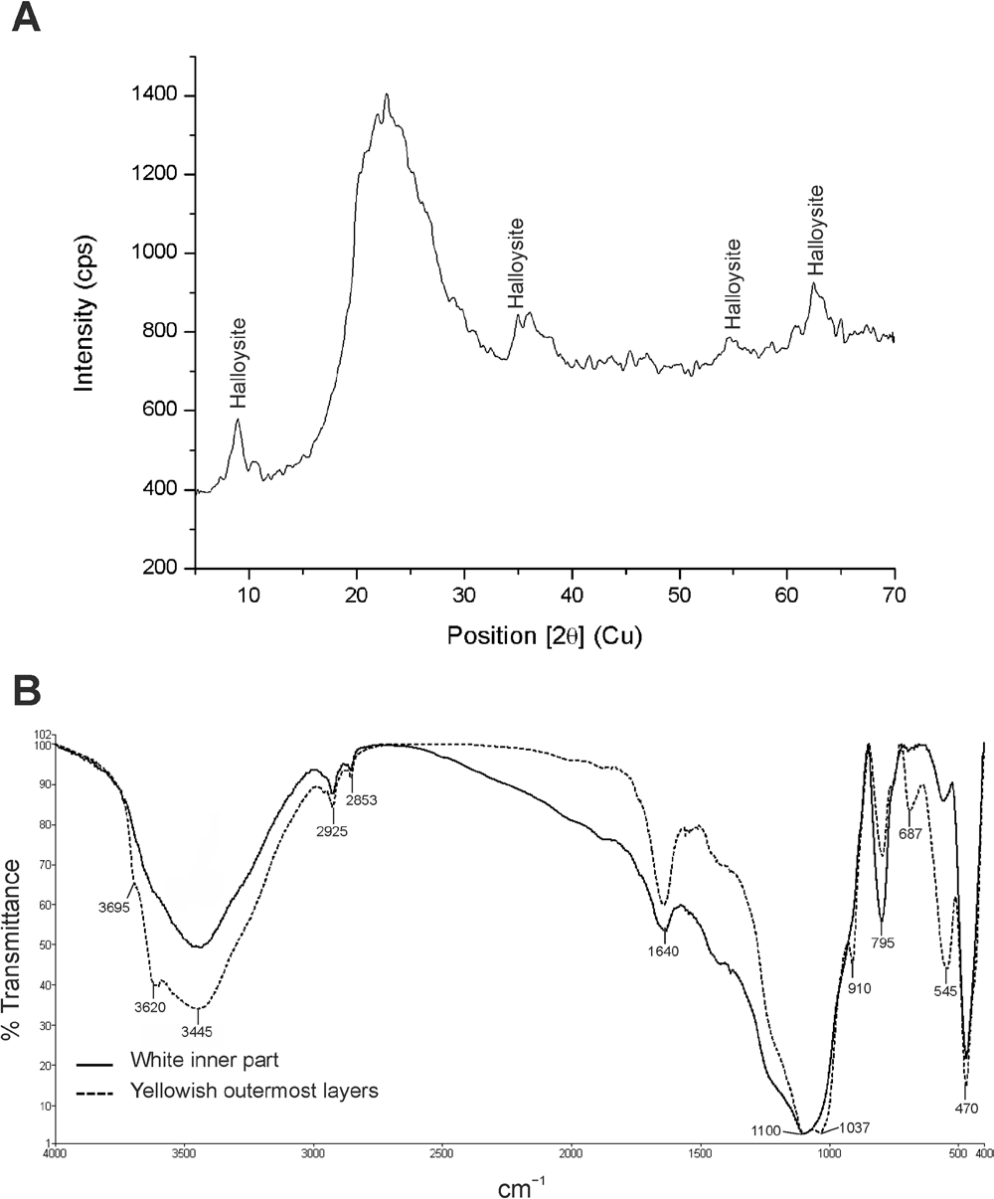
(**A**) Representative X-ray diffractogram of coralloid speleothems from EP01 lava tube showing the characteristic broad band of opal-A and peaks of hydrated halloysite. (**B**) Infrared spectra of hand-picked material.

The mineralogical analyses performed by X-ray diffraction (XRD) showed that the yellowish coralloid speleothems are composed of very low crystalline products. The XRD pattern of the coralloids displayed the typical broad diffuse band centered at about 23.4° 2*θ* (3.75 Å) of opal-A (SiO_2_.*n*H_2_0)^21,22^, with overlapped reflections attributed to hydrated halloysite, Al_2_Si_2_O_5_(OH)_4_.2H_2_O^23^ (Fig. 4A). This clay mineral was straightforwardly identified by a basal reflection at 8.9° 2*θ* (10.0 Å), and peaks centered at 20.0° 2*θ* (4.4 Å), 34.9° 2*θ* (2.57 Å), 54.6° 2*θ* (1.7 Å) and 62.4° 2*θ* (1.48 Å) (Fig. 4A), which is in good agreement with the Powder Diffraction File (PDF) card number 00-029-1489 (hydrated halloysite).

Since Fourier transform infrared (FTIR) spectroscopy is sensitive to both crystalline materials with long-range order and amorphous materials with short-range order, as well as to organic components, FTIR analysis was performed to get additional compositional information of the coralloid speleothems. FTIR analysis confirmed the XRD data, showing the dominant presence of opaline silica in the white inner part of the coralloid (Fig. 4B). The vibrational bands at 1100 cm^−1^, 795 cm^−1^ and 470 cm^−1^ were very similar to those described for opal-A^24,25^. Regardless opal, the infrared spectrum of the yellowish outermost layers showed important features attributed to halloysite in the hydroxyl stretching region (bands at 3695, 3620 and 3445 cm^−1^, Fig. 4B), at 1640 cm^−1^ corresponding to strongly hydrogen bonded water, and bands at 1037 and 910 cm^−1^ caused by the stretching vibrations of Si–O–Si and bending modes of Al–O–H^26^. In addition, the bands centered at 2925 and 2853 cm^−1^ observed for the yellowish outermost layer (Fig. 4B) is indicative of organic carbon, which is consistent with the presence of microbial mats.

Analytical pyrolysis (pyrolysis-gas chromatography/mass spectrometry) was also conducted to infer the possible origin of cave wall pigmentation. The pyrolysate of the yellow microbial mat sample produced poor pyrochromatograms likely due to the low organic carbon and the relatively high mineral contents. At 250 °C the sample yielded mainly the fatty acids palmitic and stearic, while the second shot at 450 °C produced benzene, toluene, benzaldehyde, phenol and 3,5-dimethoxy-4-hydroxy-cinnamaldehyde, the latter compound denoting a clear lignin origin (Supplementary Fig. S3). At 450 °C pyrolysis products from carbohydrate and/or polysaccharides such as furan, methylfuran, furfural, methylfurfural and levoglucosan were identified, as well as methylpyrrole, pyrroldione, benzonitrile, methylbenzonitrile and diketodipyrrole from proteins. Traces of a few sterols were evident, from which only stigmasten-3,5-diene could be recognized. This is a pyrolysis product from the plant sterol β-sitosterol^27^.

### Microbial diversity in La Palma lava tube

Molecular identification of yellow bacterial communities was determined by parallel analysis of total DNA and RNA clone libraries for the identification of metabolically active microorganisms and complemented with next-generation sequencing of the 16S rRNA gene. A total of 198 clone sequences were identified for the clone libraries, whereas 162010 reads were obtained for NGS, which were gathered into 81 and 15424 Operational Taxonomic Units (OTUs), respectively.

**Figure 5:**
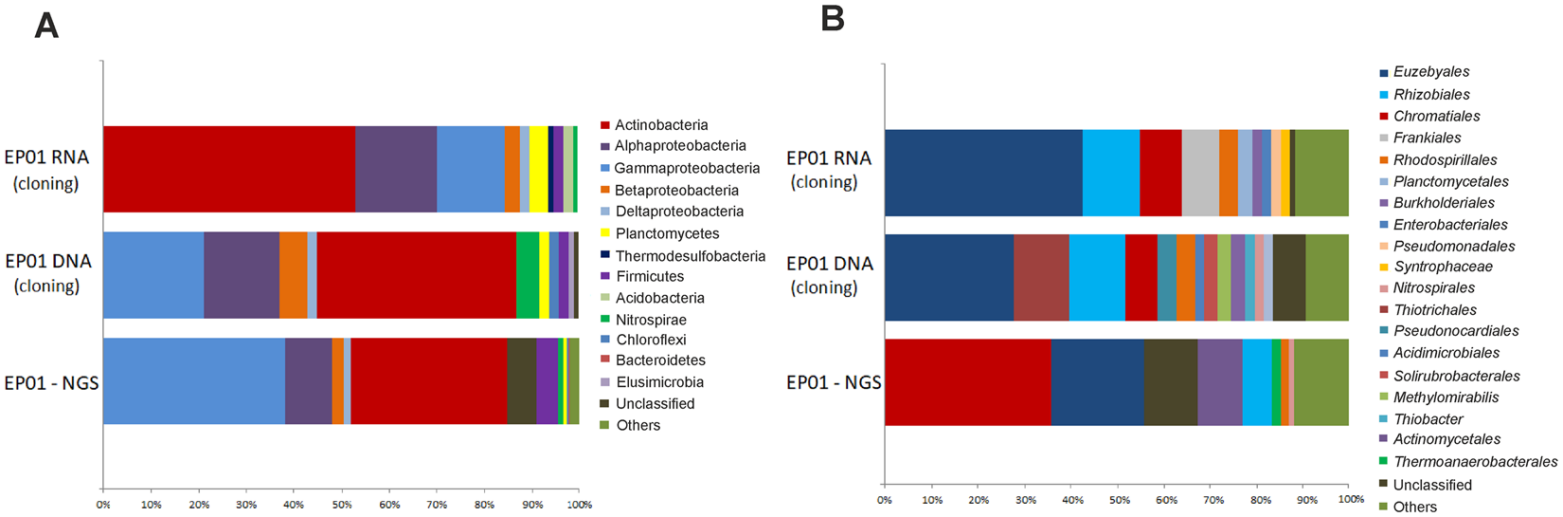
OTUs distribution in EP01 lava tube retrieved from DNA and RNA clone libraries, and NGS approach. (**A**) Phylum level. (**B**) Order level.

Clear differences were observed between DNA and RNA clone libraries (Fig. 5A). *Proteobacteria*, represented by Alpha-, Beta-, Gamma- and Delta-proteobacteria classes, was the most abundant phylum in the total bacterial community (45%), followed by *Actinobacteria* (Fig. 5A, EP01 DNA). This latter was the main metabolically active phylum, comprising 53.1% of the retrieved clones. Within the *Proteobacteria* phylum, the class *Gammaproteobacteria* was the most representative for the DNA clone library (21% EP01 DNA) except for EP01 RNA clone library, in which *Alphaproteobacteria* (17.4%) was the most predominant class. Beta- and *Deltaproteobacteria* were present in all clone libraries, but in less abundance. *Firmicutes*, *Acidobacteria*, *Thermodesulfobacteria*, *Planctomycetes*, *Nitrospirae*, *Chloroflexi*, *Elusimicrobia*, and *Bacteroidetes* were also retrieved, with amounts below 5%. The OTUs below 80% of similarity with the closest cultured relative were considered as unclassified, representing <2% of the total clones (Fig. 5).

Composition of microbial phyla obtained by NGS is also presented in Fig. 5A, revealing a similar trend with the DNA clone library, in which *Proteobacteria* was the most abundant phylum followed by *Actinobacteria*. The most common class within *Proteobacteria* was also *Gammaproteobacteria* in the NGS study. Differences between DNA clone library and NGS was mainly observed for the unclassified group, which comprised 6% of the OTUs.

Regarding the order level (Fig. 5B), *Euzebyales* represented 42.9% for the RNA clone library and 25.9% of the DNA sequences. A significant abundance was also verified for *Rhizobiales* (12.2% for RNA and 12.0% for DNA) and *Chromatiales* (10.4% for RNA and 5.3% for DNA). Yet, some OTUs were solely present in one clone library, such as *Frankiales* (8.2% RNA) and *Thiotrichales* (12% DNA) (Fig. 5B). The unclassified group increased at this taxonomic level, particularly for the DNA clone library (Fig. 5B, EP01 DNA) when compared with the phylum level (Fig. 5A). Comparison of the distribution of the identified orders generated by clone libraries with the NGS data showed some differences, with the exception of the *Euzebyales* order (20.1%). The high percentage of *Chromatiales* (35.9%) in the NGS library versus clone libraries, and the increase of unclassified OTUs were remarkable (Fig. 5B).

It is worth mentioning that clones tentatively affiliated to *Euzebya tangerina* were the most abundant in the RNA library (Supplementary Table S2). Many of the closest matches obtained in the identification of OTUs were recovered from subterranean environments, especially from lava caves in Azores, Portugal^13^ and other volcanic caves from Hawai’i and New Mexico, USA^28^ (Supplementary Tables S1–2). Indeed, three out of four closest matches identified as *Euzebyales* were retrieved from Pahoehoe Cave, New Mexico.

### Statistical evaluation of richness and diversity

The richness and diversity was assessed carrying out statistical analysis based on alpha and beta diversity. Rarefaction curves were built to calculate the expected number of species as a function of sampling effort^29^. None of the clone libraries retrieved from EP01 lava tube reached the saturation point (Supplementary Fig. S4). Richness and diversity indices were also implemented in this study. Shannon index increases with species evenness and richness, whereas the Simpson index takes into account the relative abundance of species showing values close to 0 when diversity tends towards infinity and values close to 1 if there is no diversity in the sample. Thus, the obtained Shannon and Simpson’s index values suggest a higher microbial diversity in the DNA library than in RNA (Table 1), as expected^30^. ACE and Chao 1 estimators revealed that additional cloning data are needed to cover the whole microbial community present in the studied cave (Table 1). Therefore, NGS of the 16S rRNA gene was conducted for better understanding the bacterial diversity thriving in the studied lava tube.

**Table 1:** Richness estimators for microbial community 16S rRNA libraries in this study.

Cave	Clone library	No. OTUS	No. Sequences	Richness estimation			
				ACE	Chao 1	Shannon	Simpson
EP01	DNA	46	100	352.8 (253–500.9)	158.2 (87.7–347.7)	3.2 (2.9–3.4)	0.09
	RNA	35	98	524.0 (379.8–728.6)	129.5 (67.5–310.2)	2.5 (2.2–2.8)	0.2

**Figure 6:**
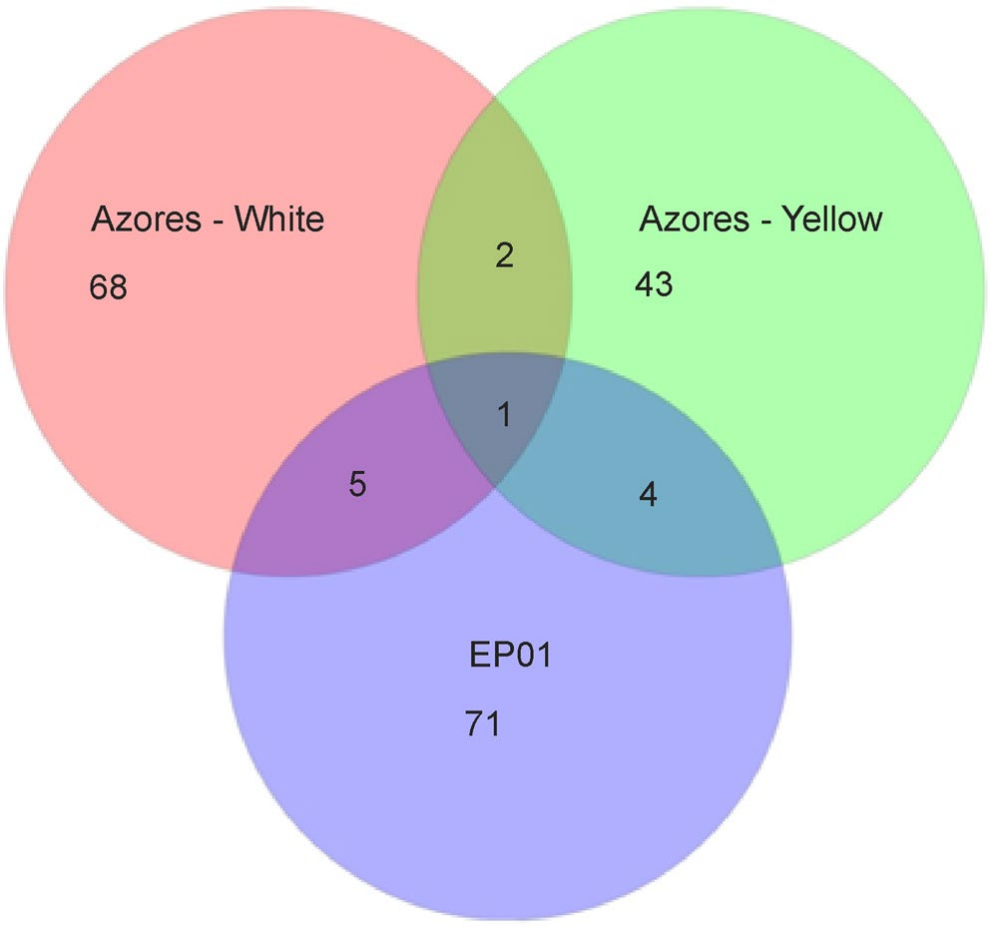
Venn diagram at a distance of 0.03 of the shared OTUs among EP01 and OTUs from yellow and white microbial mats found in Azores lava tubes studied by Riquelme *et al*.^13^.

Venn diagram of the shared OTUs between EP01 and yellow and white microbial mat sequences from Azores retrieved by Riquelme *et al*.^13^ is shown in Fig. 6. This analysis revealed higher significance between microbial communities from EP01 and Azorean lava tubes (10 shared OTUs) than between white and yellow mats from Azores (3 shared OTUs, Fig. 6). Focusing on the sample colour, there were more overlapped OTUs between EP01 and Azorean white mats, representing 7.0% of OTUs from EP01, than between EP01 and Azorean yellow colonies (only 4, representing 5.6% of the identified OTUs).

## Discussion

Yellow microbial mats covering coralloid speleothems in EP01 lava tube from La Palma Island (Spain) were investigated and compared with yellow and white microbial mats from Azores lava tubes (Portugal). Coralloid speleothems are commonly found either on the surface of basaltic lava caves or in limestone caves^22,31^. They are defined as a variety of white to grey to dark brown nodular, globular, botryoidal or coral-like speleothems^31^. Their mineralogy has been described as amorphous silica, mainly opal-A^22,32^, and their genesis has been interpreted as microbially-mediated^21,25,33^. Mineralogically, the yellowish coralloids from EP01 lava tube are almost entirely formed by amorphous opal-A and minor contribution of halloysite. The XRD patterns showed identical patterns to the standards of opal and hydrated halloysite^21–23^. The identification of halloysite was straightforward due to the presence of a basal reflection at 8.9° 2*θ*, which is due to its tubular morphology, high degree of disorder and small crystal size^26^.

The micro-CT imaging was in line with XRD and FTIR data, suggesting different water regimes during speleothem formation inducing variations in material density but homogenous mineralogical composition (Fig. 2). It is well known that the amount of water entering into a cave determines speleothem growth and composition due to water-rock interactions^22,25^. The presence of halloysite in the yellowish coralloid speleothems from La Palma is probably related to weathering processes of volcanic rocks^34,35^ and indicates that the genesis of the coralloids have a close relationship with the water-rock interactions in the EP01 lava tube. The contribution of dissolved organic matter to basalt weathering is well-known^36^ although a microbial mediation cannot be neglected^37^. The potential role of weathered basalt in enhancing bacterial growth was demonstrated by Daughney *et al*.^38^ and Sudek *et al*.^39^.

The actinobacteria-like structures observed by FESEM on the surface of the coralloids have been frequently reported in limestone and volcanic subsurface environments, as well as in soils, marine and extreme arid environments^5,13,14,28^. The morphologies of the yellow colonies from EP01 lava tube resemble those reported by Cuezva *et al*.^40^ from Altamira Cave (Spain) and Riquelme *et al*.^13^ from several lava tubes worldwide, which were mainly colonized by *Actinobacteria*. It is well known that actinobacteria are the common inhabitants of soils. They are an ecologically significant group, which play a key role in biogeochemical cycles and biomineralization processes, promoting mineral dissolution or the precipitation of secondary minerals^25,40^. In fact, the RNA-based study revealed that *Actinobacteria* represented the most abundant metabolically active phylum.

Previous studies have achieved a global description of microbial communities from different coloured colonizations in lava tubes from the Azores^1,5,13^. The NGS approach and DNA-clone library revealed that the predominant phylum was *Proteobacteria* (53 and 45%, respectively), which is in agreement with the results of the former studies. The identified phyla obtained in this work were also similar to those reported in karstic caves^1,4,5,17–19,41–43^. However, focusing on the RNA-clone library, *Actinobacteria* was the most abundant phylum, being *Euzebyales* the predominant order. In recent studies on lava tubes, several OTUs were affiliated to *Euzebyales*^28^, emerging as a bacterial community with relative importance in abundance in these environments. To the best of our knowledge, abundant *Euzebyales* have been observed in a North Spain karstic cave (unpublished data) but limited in other subterranean environments^30,40^. *Euzebya tangerina* is the only known species from this order, which was isolated from a sea cucumber^44^. Nearly 55% of the clones were tentatively affiliated to this species (92–93% similarity) but our data point to new species or genera from this order in the studied lava tube of La Palma. Unfortunately, there is no knowledge on its metabolic pathways and attempts to isolate this bacterium failed until now.

The microbial distribution found in La Palma lava tube is in fair agreement with the hypothesis established by Hathaway *et al*.^5^ who concluded that the distribution of microorganisms in caves is not cosmopolitan. In fact, the low number of shared OTUs between EP01 and Azorean lava tubes (10 OTUs, Fig. 6) is in line with this hypothesis. *Actinomycetales*, the most representative order of the *Actinobacteria* phylum found in the Azorean lava tubes, is absent in the EP01 DNA clone library and appear as the fourth order in abundance for the NGS results. Within *Actinobacteria*, *Euzebyales* was more abundant in EP01 than in Azores, i.e. the representativeness of members of this order is lower in this archipelago than in La Palma^28^. The differences in microbial diversity observed between the studied lava tube and Azorean lava tubes may be related to the geographical distance, altitude and external agents, such as climatic conditions, land use and composition of the overlying soil^5,13,45^. Lavoie *et al*.^14^ showed a close relation between the use of the surface soils and the presence of bacteria in lava tubes from Lava Beds National Monument (USA). In fact, EP01 is located in a protected natural ecosystem (Caldera del Taburiente National Park) and the most representative phylotype after *Euzebya* were *Methylocapsa aurea* and *Thioalkalivibrio denitrificans* according to the RNA clone library (Supplementary Table S2). The first one is a methanotroph belonging to the order *Rhizobiales* originally isolated from an undisturbed forest soil^46^ and the second one is a chemolithoautotrophic sulfur-oxidizing bacterium isolated from soda lakes^47^. Both isolation sources comprise low human impact areas. This is consistent with the activities on the top soil indicating that the soil overlying caves can be a source of bacteria. However, Lavoie *et al*.^14^ showed that only 11.2% of the OTUs overlapped between cave microbial mats and surface soil, suggesting that soil microorganisms may migrate into underlying caves but once there, environmental and chemical factors determine their survival and adaptation to these habitats.

The Shannon and Simpson indices were useful to differentiate the microbial diversity between DNA and RNA clone libraries performed in this study. Hence and as expected, microbial communities were more diverse for DNA than for RNA-clone libraries as also revealed by the rarefaction curves. The presence of OTUs with relative abundance in the DNA clone library but absent in the RNA library could be due to a latent stage of non-metabolically active communities, although relic DNA from dead bacteria, which persist on mineral surfaces for years cannot be discarded^48^. The comparison of DNA-RNA libraries has been previously performed with the aim of having a deeper knowledge on the biogeochemistry of subsurface environments^17,30,42,49^. In this sense, the most representative orders retrieved in the RNA-clone library of La Palma lava tube, *Euzybiales*, *Rhizobiales* and *Chromatiales* are active bacteria probably involved in cave biogeochemical processes.

The NGS approach allowed a more complete picture of the total microbial community within EP01 lava tube because it produces great sequencing depth instead of few hundred clones. Thus, at the phylum level the NGS data reinforced the diversity distribution generated by clone-based sequencing. However, at the order level the most abundant taxonomic group was *Chromatiales* instead of *Euzebyales*. One of the drawbacks of the NGS approach related to taxonomic classification at lower levels is the shorter read length of NGS technologies^50–52^. In addition, when comparing results across platforms, one of the most important bias of the 16S PCR-based sequencing (Sanger or NGS) is the choice of the hypervariable region to be analysed. The use of different primer sequences, targeting different regions of the 16S rDNA, can generate completely different results^53^. In our analyses, we performed clone libraries using the primers 616F and 1510R, which target the regions V4-V9, whereas the NGS analyses were performed using the standard primers of Illumina, targeting the regions V3-V4. Even if the V4 region was analysed with both strategies, the other regions (V3 and V5-V9) may be the reason for the different phylogenetic distributions.

The origin of microbial mats colouration has been discussed and assigned to different taxonomic groups by several authors^4,14,17,18^. Porca *et al*.^4^ showed that the yellow colonizations from several karstic caves are composed of *Pseudonocardiaceae*, *Chromatiales* and the genus *Nitrospira*, whereas Lavoie *et al*.^14^ suggested that yellow coloured mats may be produced by *Actinobacteria*. Considering that *Actinobacteria* were the most representative metabolically active phylum found in La Palma lava tube, these bacteria could be responsible for the yellow pigmentation of the cave walls if we consider that the presence of pigmentation come up from the most abundant phylogenetic groups. However and comparing both studies, there is no similarity between the phylotypes associated with *Actinobacteria*. In fact, the nature and origin of wall pigmentation in lava tubes is a contradictory issue. For instance and as mentioned before, the overlap in shared OTUs was greater between the yellow mats from EP01 and the white colonizations from Azores. This evidences that the hypothesis about whether coloured mats are caused by several particular phylotypes of bacteria is not unanimous. In fact, Hathaway *et al*.^5^ and Riquelme *et al*.^13^ pointed out that the origin of wall pigmentation in lava tubes is caused by the activity of fungi and/or archaea. Indeed, Jaspers and Overmann^54^ observed that identical 16S rRNA gene sequences could be found in bacteria with divergent genomes. So, the colouration in lava tube walls could be originated by the same phylotype with different metabolic activity or divergent phylotypes with identical metabolic pathways.

Water percolates down through the overlying basaltic rock increasing basalts weathering and the formation of coralloid speleothems. This water also contains dissolved organic matter, mainly coming from lignin degradation as observed in other caves^9^. The identification of 3,5-dimethoxy-4-hydroxy-cinnamaldehyde by analytical pyrolysis, a yellow phenolic compound, in the pyrolysate of the yellow microbial mat evidences this assertion and it is hypothesized that lignin phenols could also contribute to the colour observed on the lava tubes walls. In general, most of the compounds identified by analytical pyrolysis are common thermal evaporation/degradation products of lipids, carbohydrate and proteins either in microorganisms or plants^27,55^. The cinnamaldehyde and β-sitosterol derivatives point to a direct plant origin^27,56^.

To conclude, comparison between DNA- and RNA-based analyses and NGS approach led us to determine the most abundant phylotypes inhabiting a lava tube of La Palma Island. The phylum *Actinobacteria*, and more specifically, the order *Euzebyales*, was the most representative and metabolically active phylogenetic group for the studied cave. The diversity observed in the yellow mats is not in agreement with the phylotypes reported in previous studies as responsible for the yellow pigmentation of cave walls. However, we cannot assign the colouration of the microbial mats to a specific phylotype due to the fact that members of the order *Euzebyales* represented 100% of the RNA-based community from pink coloured mats in a North Spain cave (unpublished data). Relative distance and geographical barriers may suppose a determinant factor in bacterial abundance. Finally, a deeper study must be developed to determine the role of bacteria, fungi and/or archaea in lava tube pigmentation, as suggested in previous studies^5,13^.

## Methods

### Sampling site description and sample collection

The Island of La Palma (28°40′0″N, 17°52′0″W) belongs to the “Canary archipelago”, located in the Macaronesia region along with Madeira, Azores and Savage Islands (Portugal) and Cape Verde. Yellow microbial mats were collected from *Honda del Bejenado* (EP01) Cave (Supplementary Fig. S1). This lava tube is located in the Caldera del Taburiente National Park at 1180 m of altitude, is 1363 m in length and is covered by a conifer forest at the surface. The average area pluviometry is 423 mm/year. The age of this cave is around 700.000–750.000 years^57^. Lava compositions range from basanite to phonolite with titaniferous clinopyroxene, olivine and kaersutite as the major phenocryst phases in the mafic rocks^58^.

Samples of yellow microbial mats were gently scraped from the substrate using a sterile scalpel and gathered into sterile Eppendorf tubes. The samples were preserved at 4 °C until arrival at the laboratory and then stored at −80 °C.

### Stereomicroscopy and polished cross-section preparation

A Nikon SMZ645 stereomicroscope (200X maximum magnification) coupled with a MOTICAM 10.0 system were used to perform detailed observations of the coralloid surface and cross-sections. Polished cross-sections were obtained using a Struers Pedemin machine, an electric motor driven specimen mover, used in conjunction with the DAP-7 for grinding and polishing operations of encapsulated specimens. A Polyfast conductive resin was used to impregnate the coralloid samples.

### Field emission scanning electron microscopy

The morphology of the yellow microbial mats from EP01 lava tube was studied by field emission scanning electron microscopy (FESEM) combined with energy dispersive X-ray spectroscopy (EDS). Air-dried samples were directly mounted on sample stubs, sputter coated with gold-palladium, and subsequently examined in a Jeol JSM-7001F microscope (Jeol, Tokyo, Japan) equipped with an Oxford EDS detector (Oxford Microbeam, Oxford, UK). FESEM examinations were operated using the secondary electron (SE) detection mode with an acceleration voltage of 15 kV.

### Micro-computed tomography (µ-CT)

X-ray micro-computed tomography is a non-destructive methodology allowing a three-dimensional (3-D) observation of the samples without sample preparation. This non-destructive technique has become a standard tool for volumetric mineralogical properties of the whole volume of a solid sample. Digital radiographs were acquired with a µ-CT SkyScan 1172 (Brucker) instrument using an X-ray cone incident on a rotating specimen. The instrument comprehends a 1.3 Megapixel camera and can reach spatial resolutions of 5 µm with a detail detectability of 2 µm. The maximum object diameter is 20 mm for standard operation and 37 mm with a camera offset. Experimental conditions were optimized for the studied specimen. Maximum source power (10 W), with source voltage of 80 kV and current of 124 µA were used. Downstream 0.5 mm aluminium filtration was used to increase beam penetration in the sample to prevent “beam hardening”, a nonlinear X-ray absorption effect. The acquisition was performed by rotating the specimen over 180° with 0.7° rotational step. The pixel size (9.41 µm) was chosen in order to include the whole coralloid sample. Slice reconstructions have been obtained with the NRecon 1.6.3 routine and volumetric visualization has been achieved with DataView and CTvox programs, which integrate the instrument software packages (Brucker®). The CTvox allows the 3-D virtual visualization (image or video) of the samples.

### X-ray diffraction (XRD)

A PANalytical X’pert Plus diffractometer with Cu anode material was used to characterize the mineralogy of the coralloids according to the random powder methodology. The generator settings are 35 mA and 40 kV. The scanning ranges from 5 to 70° 2θ, with a step size of 0.05° 2θ and a scan step time of 100S. The PDF2 data base was used to identify the crystalline phases.

### Fourier Transform Infrared Spectroscopy (FTIR)

A Perkin Elmer Spectrum 65 was used to identify the functional groups of the compounds present in the two coloured parts of the coralloid samples. Approximately 1 mg of hand-picked sample material was ground and dispersed in KBr pellets. The scanning was performed between 4000 and 400 cm^−1^ with a 2 cm^−1^ resolution. This technique provides additional information concerning organic and low crystalline or amorphous phases.

### Analytical pyrolysis

Pyrolysis-gas chromatography/mass spectrometry (Py-GC/MS) was performed using a double-shot pyrolyser (Frontier Labs. model 2020i) attached to a GC/MS system Agilent 6890 N, as described elsewhere^59^. Briefly, dry samples (1 mg) were introduced into a preheated (250 °C) micro-furnace and then pyrolysed at 450 °C. The compounds evolved were then directly injected into the GC/MS for analysis. The gas chromatograph was equipped with a HP-5ms-UI capillary column. The detector consisted of an Agilent 5973 mass selective detector, and mass spectra were acquired at 70 eV ionizing energy. Compound assignments were achieved by single-ion monitoring (SIM) and by comparison with mass spectra libraries (NIST11 and Wiley7).

### Nucleic acid extraction and amplification

Genomic DNA and RNA were extracted using the PowerBiofilm DNA and RNA isolation kits according to manufacturer’s protocol (MoBio) and quantified using a Qubit 2.0 fluorometer (Invitrogen). DNA and RNA were stored at −80 °C until their use. Extracted RNA was transcribed to complementary DNA (cDNA) using the Superscript II Reverse Transcriptase (Invitrogen). The 16S rRNA gene specific primer 1510R (5′-GGCTACCTTGTTACGACTT-3′)^60^ was used during reverse transcription.

Amplification of 16S rDNA and 16S rRNA (via cDNA) was performed by Polymerase Chain Reaction (PCR) with the bacteria-specific primers 616F (5′-AGAGTTTGATYMTGGCTCAG-3′)^61^ and 1510R. PCR reactions were performed in a Bio-Rad iCycler thermal cycler (Bio-Rad). The PCR reaction mixture (1 ml) consisted of 775 µl of sterile ultrapure water, 200 µl of PCR buffer (MyTaqTM DNA Polymerase, BIOLINE), 5 μl of Taq polymerase (MyTaqTM DNA Polymerase, BIOLINE) and 10 μl of each primer (50 mM). PCR reactions were performed in 0.2 ml PCR tubes containing 25 or 50 μl of reaction mixture and from 0.5 to 2 μl of DNA template (pure or diluted to 2 and 5 ng/μl). PCR amplifications were performed using the following thermal conditions: 94 °C for 2 min; 35 cycles of 94 °C for 20S, 55 °C for 20S (50 °C for ITS regions), 72 °C for 2 min; and a final step of 72 °C for 10 min. Positive and negative controls were included in all amplification experiments. All amplified products were purified with High Pure PCR Purification Kit (Roche) according to the manufacturer’s protocol and stored at −20 °C for further analysis.

### Clone library construction

In order to obtain information of the major bacterial members, 16S rRNA gene libraries were constructed with the TOPO TA Cloning kit (Invitrogen). Transformants were randomly picked after incubation overnight at 37 °C and transferred to multi-well plates containing Luria–Bertani (LB) medium supplemented with 100 μg·ml^−1^ ampicillin. Afterwards the plates were incubated overnight at the same temperature. Amplification of plasmids for confirming the presence of inserts was carried out using the primer pair M13/T7 (5′-CAGCAAACAGCTATGAC-3′/5′-TAATACGACTCACTATAGGG-3′). On average 100 clones from a total of two libraries for DNA and RNA were sequenced by Macrogen Europe Sequencing Services (The Netherlands) using the universal bacterial primers 616F and 1510R.

### Sequence comparison and statistical data analyses

Mothur platform^62,63^ was used to carry out taxonomic classification and statistical data analysis. Sequences were checked for chimera using chimera.slayer implemented in mothur. Putative chimeric sequences were excluded from further analysis. After quality control, sequences were aligned and assigned to operational taxonomic units (OTUs) and defined at 97% identity. Taxonomic classification was done by comparing the sequences to the non-redundant database of the National Center for Biotechnology Information (NCBI) using the BLASTN algorithm, and EzBioCloud database^64^. Statistical data analysis relied on calculating rarefaction curves and community diversity indices (Chao1, Shannon and Simpson).

Clone sequences were deposited in the NCBI GenBank database (http://www.ncbi.nlm.nih.gov/genbank/) with accession numbers from LT854948 to LT855028. For comparison purposes, sequences retrieved from yellow and white communities in Azorean lava tubes (Portugal) was carried out using sequences retrieved by Riquelme *et al*.^13^. Accession numbers corresponding to this study are given in Supplementary Information (Supplementary Tables S1,S2).

### Next-generation sequencing and data analysis

The extracted DNA (with a concentration of 242 ng/µl) was analyzed with next-generation sequencing (NGS) of the bacterial V3 and V4 regions of the 16S SSU rRNA gene using Illumina MiSeq. 2 × 300 paired end sequencing by STAB Vida sequencing services (Portugal). Raw data was processed in QIIME 1.9.1^65^. First, paired end reads were assembled using PEAR^66^. Quality control and trimming were performed using FASTQC (http://www.bioinformatics.babraham.ac.uk/projects/fastqc/) and bbduk (minlength = 75 trimq = 20; http://jgi.doe.gov/data-and-tools/bb-tools/). Operational Taxonomic Units (OTUs) were clustered with a 97% cutoff using UCLUST^67^. The National Center for Biotechnology Information (NCBI) bacterial database was used for assign the taxonomy classification of each 16S rRNA gene sequence with a threshold of 90%. Chimeric sequences were detected and removed using USEARCH. Alpha_diversity.py command was employed for alpha diversity measurements. Sequences were registered and deposited in the Sequence Read Archive (SRA) database of the NCBI with accession number SAMN07828193.

### Accession codes

Clone sequences were deposited at GenBank under the accession numbers LT854948-LT855028. NGS sequences were registered and deposited in the Sequence Read Archive (SRA) database of the NCBI with accession number SAMN07828193.

## Electronic supplementary material

Supplementary Information
