# Supplementary material for: Yellow coloured mats from lava tubes of La Palma (Canary Islands, Spain) are dominated by metabolically active Actinobacteria
Source: Sci Rep. 2018 Jan 31;8:1944. doi: 10.1038/s41598-018-20393-2 (PMC5792456; doi:10.1038/s41598-018-20393-2)
Supplement: Supplementary file 1 — Supplementary Information [file 41598_2018_20393_MOESM1_ESM.pdf]

Supplementary Information

Yellow coloured mats from lava tubes of La Palma (Canary Islands, Spain) are dominated by metabolically active Actinobacteria

Jose L. Gonzalez-Pimentel[1], Ana Z. Miller[1]*, Valme Jurado[1], Leonila Laiz[1], Manuel F.C. Pereira[2], Cesareo Saiz-Jimenez[1]

[1]Instituto de Recursos Naturales y Agrobiologia de Sevilla (IRNAS-CSIC), Avenida. Reina Mercedes 10, 41012 Sevilla, Spain.

[2]CERENA, Instituto Superior Técnico, Universidade de Lisboa, Avenida. Rovisco Pais, 1, 1049-001, Lisbon, Portugal

*corresponding author: anamiller@irnas.csic.es

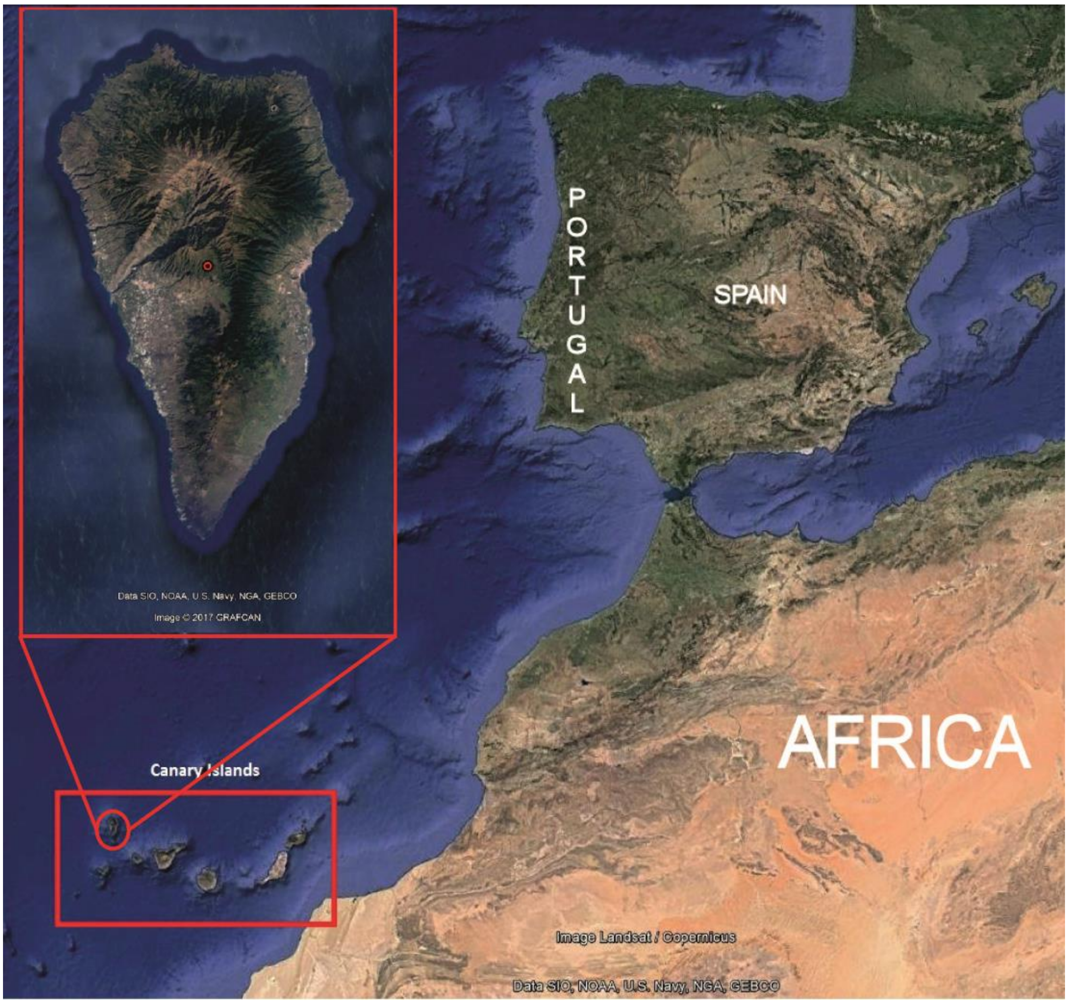

Figure S1. Studied lava tube in La Palma (EP01). Geographical location of Canary Islands (Spain) in the Atlantic Ocean and in La Palma. Source: Google Maps. (2017). *La Palma, Canary Islands*. Retrieved from https://goo.gl/maps/wSZxPcRYihM2

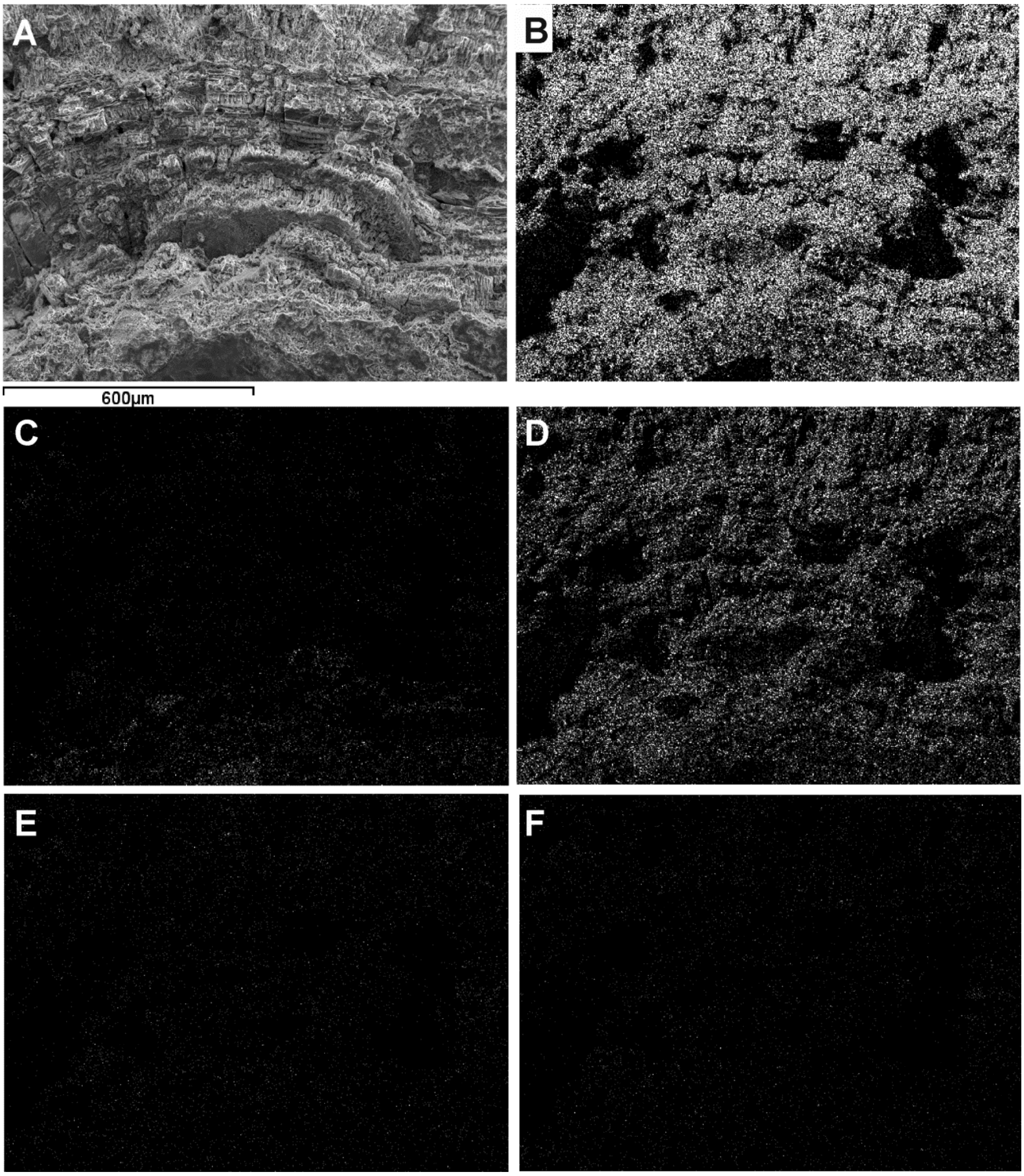

Figure S2. FESEM-EDS analysis of a polished cross-section from a coralloid speleothem of EP01 lava tube in La Palma. (A) FESEM image of the selected area. (B) Elemental map of the selected area showing the distribution of Silicon. (C) Elemental distribution map of Carbon. (D) Elemental distribution map of Oxygen. (E) Elemental distribution map of Aluminium. (C) Elemental distribution map of Magnesium.

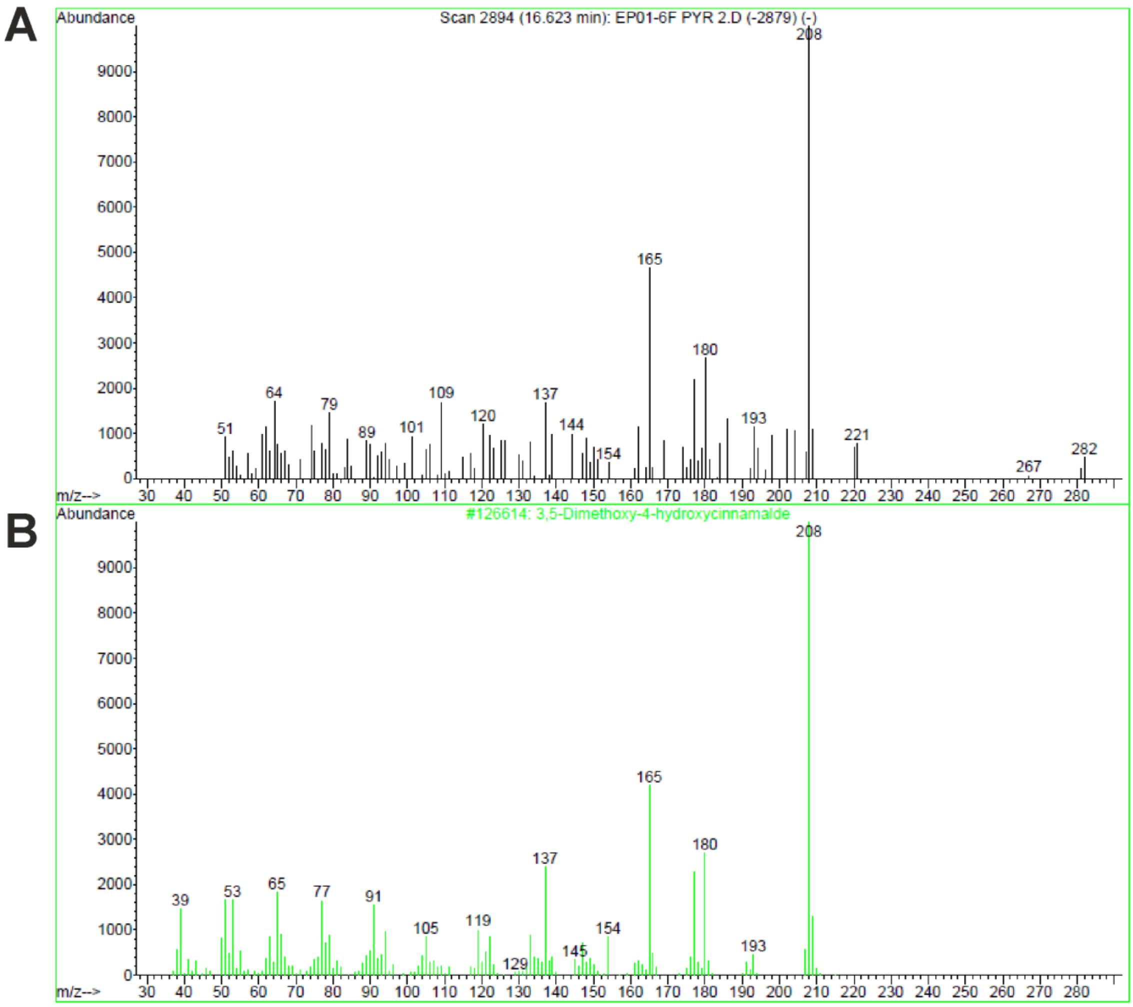

Figure S3. Mass spectra of 3,5-dimethoxy-4-hydroxycinnamaldehyde evolved from the pyrolysis of the microbial mats from EP01 lava tube (A) and Wiley library (B).

A

B

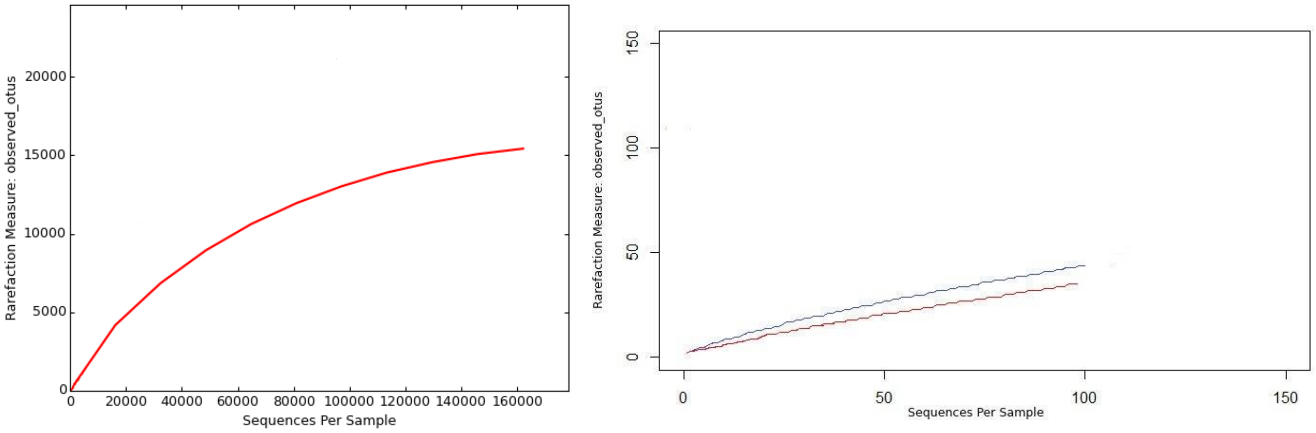

Figure S4. Rarefaction curves. (A) EP01 DNA NGS library. (B) EP01 DNA (Blue) and RNA clone libraries (Red) based-OTUs.

Table S1. Phylogenetic affiliations of the 16S rRNA sequences obtained from total bacteria in EP01 sample.

Representative clone (Accession number)	No. of Clones	Nearest relative and source	Similarity	EzBioCloud affiliation (accession number)	Similarity
LT854957	27	Uncultured bacterium clone from white microbial mat from lava tube in USA: Pahoehoe Cave, New Mexico. (KC331729)	99%	*Euzebya tangerina* (AB478418)	92.52%
LT854955	10	Uncultured bacterium clone from cave wall biofilm, Slovenia	99%	*Thiophysa hinzei* (FR690984)	90.99%
LT854948	5	Uncultured bacterium clone from white microbial mat from lava tube in Portugal: Gruta das Torres, Pico, Azores (JF266429)	99%	*Methylocapsa aurea* (JQKO01000009)	97.43%
LT854991	4	Uncultured bacterium clone from lava tube wall. Portugal: Gruta da Balcoes, Terceira, Azores (JN672330)	99%	*Thioalkalivibrio denitrificans* (AF126545)	92.93%
LT854984	3	Uncultured bacterium clone from lava tube wall. Portugal: Gruta da Madre de Deus, Terceira, Azores. (JN615883)	100%	*Methyloceanibacter caenitepidi* (AB794104)	96.14%
LT854959	3	Uncultured bacterium clone from soil of grass prairie (EU132508)	97%	*Solirubrobacter phytolaccae* (KF459924)	91.58%
LT854983	3	Uncultured bacterium clone from deposit. Hungary: Molnar Janos thermal karst cave (LN998925)	100%	*Methyloceanibacter caenitepidi* (AB794104)	96%
LT854977	3	Uncultured bacterium clone from white microbial mat from lava tube in USA: Roots Galore Cave, New Mexico. (KC331797)	99%	*Pseudonocardia bannensis* (FJ817375)	94.45%
LT854956	2	Uncultured bacterium clone in cave wall biofilm, Czech republic (HE602897)	99%	*Thiophysa hinzei* (FR690984)	91.16%
LT854975	2	Uncultured bacterium clone from subsurface aquifer sediment (JX221819)	100%	*Methylomirabilis oxyfera* (FP565575)	87.45%
LT854986	2	Uncultured bacterium clone from cave wall biofilms from the Frasassi cave system, Italy (DQ499328)	100%	*Thiobacter subterraneus* (AB180657)	91.75%
LT854951	2	Uncultured bacterium clone from white microbial mat from lava tube. Portugal: Gruta do Natal, Terceira, Azores (JN672462)	99%	*Glaciimonas immobilis* (GU441679)	91.73%
LT854972	1	Uncultured bacterium clone from soil of Yanshan Mountain (KC554485)	99%	*Streptacidiphilus neutrinimicus* (AF074410)	88.63%
LT854976	1	Uncultured bacterium isolate from soil from a natural CO2 spring (HF952279)	98%	*Gaiella occulta* (JF423906)	91.83%
LT854954	1	Uncultured bacterium clone from yellow microbial mat from lava tube wall. Portugal: Azores, Gruta Madre de Deus Terceira. (JN701063)	99%	*Sphaeronema italicum* (AY428765)	91.37%
LT854973	1	Uncultured bacterium clone from microbial mat from lava tube wall. Portugal: Azores, Gruta Madre de Deus Terceira. (JN672491)	99%	*Methylomirabilis oxyfera* (FP565575)	87.58%
LT854949	1	Uncultured bacterium clone from white microbial mat lava tube wall. Portugal: Gruta do Natal, Terceira, Azores. (JN672413)	98%	*Filomicrobium fusiforme* (Y14313)	92.98%

Table S1. Continued.

Representative clone (Accession number)	No. of Clones	Nearest relative and source	Similarity	EzBioCloud affiliation (accession number)	Similarity
LT854953	1	Uncultured bacterium clone from white microbial mat lava tube wall. Portugal: Gruta do Natal, Terceira, Azores (JN672480)	99%	*Endoriftia persephone* (AFOC01000137)	84.68%
LT854970	1	Uncultured bacterium clone from primary forest soil (EU881113)	99%	*Phaselicystis flava* (EU545827)	86.38%
LT854967	1	Micromonospora from marine sediment (HQ877445)	91%	*Micromonospora equina strain Y22* (JF912511)	91.81%
LT854966	1	Uncultured bacterium isolate from loamy sand (HQ121112)	99%	*Elstera litoralis* (LAJY01000856)	89.41%
LT854965	1	Uncultured bacterium clone from hyaloclastite deposit (GU219793)	96%	*Litorilinea aerophila* (JQ733906)	82.22%
LT854964	1	Uncultured bacterium clone from Subsurface core samples Hanford formation (HM185982)	99%	*Aciditerrimonas ferrireducens* (AB517669)	83.29%
LT854963	1	Uncultured bacterium clone from saturated C horizon soil aggregate (EU335216)	99%	*Aciditerrimonas ferrireducens* (AB517669)	83.15%
LT854962	1	Uncultured bacterium clone from Nihewan Basin soil (KT905762)	97%	*Aciditerrimonas ferrireducens* (AB517669)	88.18%
LT854960	1	Uncultured bacterium clone from Chinese paddy field (KJ877524)	95%	*Azoarcus buckelii* (AJ315676)	88.86%
LT854974	1	Uncultured bacterium clone from pink microbial mat from lava tube wall. Portugal: Algar do Carvao, Terceira, Azores (JN672018)	92%	*Endomicrobium pyrsonymphae* (AY572024)	80.64%
LT854952	1	Uncultured bacterium clone from white microbial mat from lava tube. Portugal: Gruta do Natal, Terceira, Azores (JN672474)	99%	*Nitrospira moscoviensis* (X82558)	96.76%
LT854993	1	Pseudonocardia spinosispora from Korean soil (GU318369)	97%	*Pseudonocardia spinosispora* (AJ249206)	96.67%
LT854992	1	Uncultured bacterium clone from lava tube wall. Portugal: Gruta da Balcoes, Terceira, Azores (JN672330)	99%	*Thioalkalivibrio denitrificans* (AF126545)	92.93%
LT854990	1	Uncultured bacterium clone from lava tube wall. Portugal: Gruta da Balcoes, Terceira, Azores (JN672330)	98%	*Thioalkalivibrio denitrificans* (AF126545)	92.93%
LT854971	1	Uncultured bacterium clone from yellow mat in lava tube wall. Portugal: Gruta dos Principiantes, Terceira, Azores (JN850448)	99%	*Thioalkalivibrio denitrificans* (AF126545)	92.66%
LT854989	1	Uncultured actinobacterium clone from semiarid desert soil (JQ071677)	97%	*Aciditerrimonas ferrireducens* (AB517669)	94.27%
LT854969	1	Uncultured bacterium clone from yellow microbial mat from lava tube wall. Portugal: Algar do Carvao, Terceira, Azores (JF265891)	93%	*Algisphaera agarilytica* (AB845176)	78.83%
LT854950	1	Uncultured bacterium clone from white microbial mat from lava tube. Portugal: Gruta do Natal, Terceira, Azores (JN672417)	96%	*Tistrella bauzanensis* (GQ240228)	89.74%
LT854988	1	Uncultured bacterium clone from microbial mat from lava cave wall. Portugal: Furna do Lemos, Pico, Azores (JN801132)	99%	*Nitrospira moscoviensis* (X82558)	95.81%

Table S1. Continued.

Representative clone (Accession number)	No. of Clones	Nearest relative and source (accession number)	Similarity	EzBioCloud affiliation (accession number)	Similarity
LT854968	1	Uncultured bacterium clone from yellow microbial mat from lava tube wall. Portugal: Gruta dos Montanheiros, Pico, Azores. (JF265891)	99%	*Desulfacinum hydrothermale* (AF170417)	85.09%
LT854987	1	Uncultured bacterium from rice paddy soil (AB656921)	96%	*Azospirillum halopraeferens* (Z29618)	91.16%
LT854985	1	Uncultured bacterium clone from Subsurface core samples Hanford formation (HM187359)	96%	*Thermanaeromonas toyohensis* (AB062280)	83.70%
LT854982	1	Bacillus mycoides strain ISR_8 from rhizopshere of Salicornia europaea L. (KX035066)	99%	*Bacillus mycoides DSM 2048* (ACMU01000002)	99.57%
LT854981	1	Uncultured bacterium clone from groundwater from tectonically-formed cavern (KC358408)	94%	*Blastopirellula cremea* (JF748733)	87.73%
LT854961	1	Uncultured bacterium clone from cave. Bulgaria:Northwest Bulgaria (HE653873)	99%	*Povalibacter uvarum* (AB548216)	92.42%
LT854980	1	Uncultured Rhodopirellula sp. clone UV-2_11 water from medium-term experimental oligotrophic mesocosms in Cuatro Cienegas (JQ700600)	98%	*Pirellula staleyi* (CP001848)	90.39%
LT854979	1	Uncultured bacterium clone from soil of grass prairie (EU134007)	95%	*Anaerolinea thermolimosa* (AB109437)	82.19%
LT854978	1	Uncultured bacterium clone from Subsurface core samples Hanford formation (HM186127)	97%	*Xenophilus azovorans* (JQKD01000107)	89.24%
LT854958	1	Uncultured actinobacterium from alcarenite walls of underground tombs from Roman Necropolis of Carmona (FN297998)	99%	*Euzebya tangerina* (AB478418)	92.66%

Table S2. Phylogenetic affiliations of the 16S rRNA sequences obtained from metabolically active bacteria in EP01 sample.

Representative clone (Accession number)	No. of Clones	Nearest relative and source (accession number)	Similarity	EzBioCloud affiliation (accession number)	Similarity
LT854996	42	Uncultured bacterium clone from white microbial mat from lava tube in USA: Pahoehoe Cave, New Mexico. (KC331729)	99%	*Euzebya tangerina* (AB478418)	92.97%
LT854995	8	Uncultured bacterium clone from microbial mat lava tube wall. Portugal: Furna do Lemos, Pico, Azores (JN801129)	99%	*Methylocapsa aurea* (JQKO01000009)	97.59%
LT855003	7	Uncultured bacterium clone from white microbial mat lava tube wall. Portugal: Gruta das Torres, Pico, Azores (JF266446)	99%	*Thioalkalivibrio denitrificans* (AF126545)	92.73%
LT855006	7	Uncultured bacterium clone from microbial mat from lava cave wall. Portugal: Gruta da Madre de Deus, Terceira, Azores. (JF266307)	100%	*Frankia alni str. ACN14A* (CT573213)	95.19%
LT855000	2	Uncultured bacterium clone from white microbial mat lava tube wall. Portugal: Gruta da Ribeira do Fundo, Pico, Azores. (JF266307)	98%	*Oceanibaculum pacificum* (FJ463255)	89.97%
LT855025	2	Uncultured bacterium clone from beetle gut content (KM242560)	99%	*Serratia myotis* (KJ739884)	98.43%
LT854997	2	Uncultured bacterium clone from white microbial mat lava tube wall. Portugal: Gruta das Torres, Pico, Azores (JF266456)	99%	*Thioalkalivibrio denitrificans* (AF126545)	92.73%
LT855016	1	Uncultured bacterium clone from soil of grass prairie (EU135153)	98%	*Gemmata obscuriglobus* (ABGO01000325)	87.30%
LT855026	1	Uncultured bacterium clone from yellow microbial mat from lava tube walls. Portugal: Azores, Terceira, Gruta da Achada Lava Tube. (HM445006)	95%	*Thermosulfurimonas dismutans* (JF346116)	84.05
LT855004	1	Uncultured bacterium clone from white microbial mat lava tube wall. Portugal: Gruta do Natal, Terceira, Azores. (JN672384)	100%	*Acidothermus cellulolyticus* (CP000481)	85.66%
LT855014	1	Uncultured bacterium clone from butterscotch organic ooze from lava tube wall. Portugal: Gruta dos Principiantes, Terceira, Azores. (JN616120)	99%	*Gaiella occulta* (JF423906)	93.53%
LT855013	1	*Pseudomonas chlororaphis* subsp. Aurantiaca from a wheat head (KF879088)	99%	*Pseudomonas chlororaphis subsp. Piscium* (FJ168539)	99.86%
LT855012	1	Uncultured *Gemmata* sp. clone from *Pinus massoniana* soil (KJ192111)	96%	*Telmatocola sphagniphila* (JN880417)	85.93%
LT855011	1	Uncultured bacterium clone from subsurface thermal spring (FR863662)	95%	*Telmatocola sphagniphila* (JN880417)	85.20%
LT855010	1	Uncultured bacterium clone from Leaf-cutter ant refuse dumps (LN573571)	95%	*Anoxynatronum sibiricum* (AF522323)	86.25%
LT855009	1	Uncultured bacterium clone from undisturbed tall grass prairie (FJ479291)	99%	*Syntrophus aciditrophicus* (CP000252)	86.86%

Table S2. Continued.

Representative clone (Accession number)	No. of Clones	Nearest relative and source (accession number)	Similarity	EzBioCloud affiliation (accession number)	Similarity
LT855008	1	Uncultured bacterium clone from loess (GQ214090)	98%	*Syntrophus aciditrophicus* (CP000252)	86.75%
LT855007	1	Uncultured bacterium clone from anaerobic digester of a wastewater plant (CU925538)	97%	*Bythopirellula goksoyri* (KF607112)	96.42%
LT854999	1	Uncultured bacterium clone from microbial mat from lava cave wall. Portugal: Gruta da Madre de Deus, Terceira, Azores. (JN615923)	99%	*Nakamurella flavida* (DQ321750)	94.41%
LT854994	1	Uncultured bacterium clone from pink microbial mat from lava tube wall. Portugal: Algar do Carvao, Terceira, Azores (JN672033)	99%	*Phenylobacterium koreense* (AB166881)	89.83%
LT855015	1	Uncultured bacterium clone from yellow microbial mat from lava tube wall. Portugal: Gruta do Natal, Terceira, Azores. (JN592694)	99%	*Filomicrobium fusiforme* (Y14313)	95.17%
LT854998	1	Uncultured bacterium clone from white microbial mat lava tube wall. Portugal: Gruta da Ribeira do Fundo, Pico, Azores. (JF266307)	95%	*Skermanella stibiiresistens* (HQ315828)	90.13%
LT855027	1	Uncultured bacterium clone from cave wall biofilms from the Frasassi cave system, Italy (DQ499328)	99%	*Thiobacter subterraneus* (AB180657)	91.60%
LT855024	1	Uncultured bacterium clone from Carbonate Cave Pools in New Mexico (JQ675490)	99%	*Pseudomonas peli* (AM114534)	98.79%
LT855023	1	Uncultured oligocarboniphilum from purified water system (NR_117348)	99%	*Undibacterium oligocarboniphilum* (GQ379228)	99.88%
LT855022	1	Uncultured Hyphomicrobiaceae bacterium clone from Pinus massoniana soil (KJ191977)	97%	*Pedomicrobium manganicum* (X97691)	96.18%
LT855021	1	Uncultured bacterium clone from prehistoric paintings in Magura Cave, Bulgaria (HE653878)	97%	*Thermoanaerobaculum aquaticum* (JMFG01000020)	86.73%
LT855001	1	Uncultured bacterium clone from white microbial mat lava tube wall. Portugal: Gruta da Balcoes, Terceira, Azores (JN850142)	97%	*Amorphus coralli* (KB894580)	93.39%
LT855020	1	Uncultured bacterium clone from subsurface aquifer sediment (JX221790)	99%	*Paenibacillus endophyticus* (KC447384)	98.92%
LT855005	1	Uncultured bacterium clone from white microbial mat lava tube wall. Portugal: Gruta da Balcoes, Terceira, Azores (JN850142)	99%	*Methylovirgula ligni* (FM252034)	93.53%
LT855019	1	Uncultured bacterium clone from water sample. USA: Carlsbad Caverns, New Mexico Room (JQ675422)	99%	*Methylomirabilis oxyfera* (FP565575)	93.25%
LT855002	1	Uncultured bacterium clone from white microbial mat lava tube wall. Portugal: Gruta das Torres, Pico, Azores (JF266446)	98%	*Thiophysa hinzei* (FR690984)	91.28%
LT855017	1	*Variovorax ginsengisoli* from paddy soil (AB649024)	99%	*Variovorax ginsengisoli* (AB245358)	99.03%

Table S2. Continued.

Representative clone (Accession number)	No. of Clones	Nearest relative and source (accession number)	Similarity	EzBioCloud affiliation (accession number)	Similarity
LT855018	1	Uncultured bacterium clone from pink microbial mat from lava tube wall. Portugal: Algar do Carvao, Terceira, Azores (JN671995)	98%	*Aridibacter famidurans* (KF245634)	95.40%
LT855028	1	Uncultured bacterium clone from microbial mat from lava cave wall. Portugal: Gruta da Madre de Deus, Terceira, Azores. (JN615908)	99%	*Sphaeronema italicum* (AY428765)	92.23%
